# The complete genome sequencing of *Escherichia coli* isolated from a patient who visited Pediatrics People’s Hospital of Pingguo, China

**DOI:** 10.1128/mra.00020-24

**Published:** 2024-04-09

**Authors:** Lifeng Chen, Zeeshan Umar

**Affiliations:** 1 Department of Clinical Engineering and Materials Supplies, The First Affiliated Hospital, School of Medicine, Zhejiang University, Hangzhou, China; 2 Marshall Laboratory of Biomedical Engineering, School of Medicine, Shenhzen University, Shenzhen , China; Department of Biological Sciences, Wellesley College, Wellesley, Massachusetts, USA

**Keywords:** antibiotic resistance, genomes, *E. coli*

## Abstract

In this article, we present a comprehensive analysis of the genome sequence of *Escherichia coli* isolate ACESH02881hy, which has a 5,071,463-bp genome size. The strain was isolated from patient who visited the Pediatrics People’s Hospital of Pingguo, China, 2021.

## ANNOUNCEMENT

Enterotoxigenic *Escherichia coli* (ETEC) is an important enteric pathogen that causes a significant number of cases of diarrhea annually, reaching into the tens of millions. Children under 5 years of age are highly susceptible to ETEC infection, especially in regions where the disease is prevalent. In the year 2015, ETEC was accountable for an estimated 100 million instances of diarrhea, resulting in approximately 60,000 deaths (1). This manuscript presents the genome of an ETEC strain obtained from a patient who visited Pediatrics People’s Hospital. The stool sample (1.0 g) was collected and then aerobically cultured overnight at 37°C on MacConkey agar. Next day, colonies with distinct morphologies were streaked to obtain a pure culture in order to identify the bacteria using matrix-assisted laser desorption/ionization time-of-flight mass spectrometry (Bruker, Bremen, Germany), accepting score values between 2.3 and 3.0 (2). Then, the DNA was extracted following the manufacturer’s instruction through SteadyPure Bacteria Genomic DNA Extraction Kit (AG) from a culture grown overnight at 37°C, and evaluated through NanoDrop 2000 UV-visible (UV-Vis) spectrophotometer (Thermo Scientific, USA) and a Qubit version 2.0 fluorometer (Thermo Scientific) (2). The same DNA was then sequenced on Illumina, Novogene Bioinformatics Technology (Beijing, China), and Oxford Nanopore. For the short-read sequencing, DNA was fragmented by sonication to a size of 350 bp and subjected to sequencing using the Illumina novaseq 6000 platform through Nextera XT Kit (Illumina, USA). Then A-tailed fragments were then ligated with paired-end adaptors and amplified through PCR using a 500-bp insert. The PCR products were then purified through the AMPure XP system (USA), and the quality of the library was evaluated using the Agilent 5400 system (USA) and quantified through qPCR (1.5 nM). Fastp (0.23.1) was used to analyze short-read sequencing quality by discarding paired reads with adapter contamination, followed by over 10% unknown bases or more than 50% low-quality bases (Phred quality < 5) at the machine’s default parameters except where otherwise noted. For long reads, the DNA library was generated without sheering the DNA either mechanically or enzymatically through a Ligation Sequencing Kit (SQK-LSK114). Subsequently, raw reads from the PromethION were initially assessed using the MinKNOW software for real-time Quality Control (QC) metrics and processed with the Oxford Nanopore Technologies Guppy software (version 0.17.1) in high-accuracy mode to enhance base calling.

While Unicycler version 0.4.7 was used for the hybrid assembly (3), the complete genome sequence of the bacteria was then submitted to NCBI for annotation and bioinformatics analysis by NCBI Prokaryotic Genome Annotation Pipeline (6.6) (4, 5). Acquired antibiotic resistance genes in L2890hy was identified by Res-Finder 4.1. Related metrics are listed in Table 1.

**TABLE 1:** Features of the complete whole-genome sequences of Escherichia coli strain ACESH02881hy

Feature		
Long read	Number of reads	99,551.0
	Total bases	1,173,847,871.0
	Mean read length (bp)	11,791.4
	Mean read quality	13.7
	Median read length (bp)	10,761.0
	Median read quality	14.2
	N50 read length (bp)	12,979.0
Short read	Total bases	11,554,920
	Raw base (Mb)	1,006
	Raw read1 Q20 (%)	97.67
	Raw read2 Q20 (%)	96.53
	Clean base (Mb)	1,006
	Clean read1 Q20 (%)	97.67
	Clean read2 Q20 (%)	96.53
	GC (%)	47.89
Assembly	Genome size (bp)	5,071,463
	Long read coverage (×)	470
	Short-read coverage (×)	200
	GC content (%)	50.6
	N50 value	853,191
	L50 value	2
	Resistance gene (antimicrobial)	*aadA1*, *aadA2* (aminoglycoside)
		*dfrA12*, *sul2*, *sul3* (folate pathway antagonist)
		*sitABCD* (peroxide)
		*tet(A)* (tetracycline)
		*bla*_TEM-1B_, *bla*_NDM-1_ (beta-lactam)
		*qacL* (quaternary ammonium compound)
		*floR*, *cmlA1* (amphenicol)

The complete genome size of *E. coli* ACESH02881hy is 5,071,463 bp. The Guanine-Cytosine (GC) content was approximately 50.6% with an L50 value of 2. Antimicrobial resistance gene analysis showed that the whole genome contained many antimicrobial resistance genes, including *bla*_TEM-1B_ and *bla*_NDM-1_ (beta-lactam).

## Data Availability

The whole-genome sequence of *E. coli* L2881hy has been deposited in GenBank, BioProject accession number PRJNA1043826, BioSample accession number SAMN38354106, SRA number SRR26924129, and Illumina SRA number SRR27393454 .
